# Metabolic Reprogramming in Gastric Cancer Immunity Mechanisms and Therapeutic Implications

**DOI:** 10.32604/or.2026.087144

**Published:** 2026-09-14

**Authors:** Xiangyang Wang, Ying Wu, Yutong Fu, Ejakpovi Emmanuel Oghenefejiro, Zakari Shaibu, Cunxi Li, Qi Zhou, Liang Yin

**Affiliations:** 1 Department of Traditional Chinese Medicine, Affiliated People’s Hospital of Jiangsu University, Zhenjiang, China; 2 Department of Breast Surgery, Affiliated People’s Hospital of Jiangsu University, Zhenjiang, China; 3 School of Medicine, Jiangsu University, Zhenjiang, China; 4 Department of Rehabilitation, Danyang Hospital of Traditional Chinese Medicine, Danyang, China; 5 Department of Endodontics and Dental Materials, Zhenjiang Stomatological Hospital, Zhenjiang, China; 6 Department of Medical Oncology, Affiliated People’s Hospital of Jiangsu University, Zhenjiang, China

**Keywords:** Gastric cancer (GC), tumor microenvironment (TME), metabolic reprogramming, immunometabolism, immunotherapy

## Abstract

Gastric cancer (GC) remains a leading cause of global cancer mortality, with progression and therapy resistance heavily influenced by the dynamic tumor microenvironment (TME). Despite advances in surgical techniques, chemotherapy, targeted therapy, and immunotherapy, overall survival for advanced disease remains poor, underscoring the need for a deeper understanding of resistance mechanisms. A hallmark of the TME is metabolic reprogramming, which sustains tumor growth and actively shapes an immunosuppressive landscape. This review aims to detail the coordinated metabolic adaptations of GC cells, cancer-associated fibroblasts (CAFs), and immune cells within the TME, focusing on nutrient competition, immunosuppressive metabolite accumulation, and dysregulated lipid metabolism. We analyze how glucose depletion, lactate accumulation, and amino acid deprivation establish a hostile metabolic niche that impairs cytotoxic T lymphocyte (CTL) function while paradoxically supporting regulatory T cells (Tregs), M2-like tumor-associated macrophages (TAMs), and myeloid-derived suppressor cells (MDSCs). We examine four major immunosuppressive metabolic pathways, lactate, adenosine, tryptophan-kynurenine, and arginine and demonstrate their convergence on immune checkpoint upregulation, forming an integrated metabolic-immune checkpoint axis. These pathways establish a self-reinforcing immunosuppressive circuit that drives T cell exhaustion and limits immune checkpoint blockade efficacy. We highlight emerging therapeutic strategies targeting this crosstalk, including inhibitors of glycolysis, glutaminolysis, indoleamine 2,3-dioxygenase 1 (IDO1), and adenosine signaling, often combined with immunotherapy. The metabolic supply-demand mismatch explains why certain interventions can revive effector cells while potentially harming other cell types. Finally, we discuss challenges and future directions, emphasizing the need for spatially resolved metabolic profiling, biomarker-driven patient stratification, and personalized therapies to overcome metabolic immunosuppression and improve clinical outcomes in GC.

## 1 Introduction

Gastric cancer (GC) is the fifth most common cancer and the fifth leading cause of cancer death worldwide, with approximately 968,350 new cases and 659,853 deaths annually [1], and is characterized by significant genomic heterogeneity including distinct molecular subtypes such as EBV+, MSI+, CIN, and GS [2]. Despite advances in surgical techniques, chemotherapy, targeted therapy, and immunotherapy, overall survival for advanced disease remains poor, underscoring the need for a deeper understanding of resistance mechanisms [3]. The tumor microenvironment (TME) is a complex ecosystem comprising cancer cells, stromal cells (fibroblasts, endothelial cells), and a diverse array of immune cells. It is now widely accepted that the biological behavior of GC is not solely determined by cancer cell-autonomous traits but is profoundly shaped by continuous and dynamic interactions within the TME [4]. A central driver of these interactions is metabolic reprogramming, a recognized cancer hallmark [5]. This reprogramming involves comprehensive alterations in glucose, lipid, and amino acid metabolism that not only fuel tumor growth but also actively sculpt the immunosuppressive landscape, as recently comprehensively reviewed [6,7]. While initially described as a means for cancer cells to support their own rapid proliferation and survival (the Warburg effect), it is now clear that metabolic alterations extend across the entire TME. Cancer cells and stromal components engage in a fierce competition for limited nutrients and simultaneously release metabolic waste products that potently modulate immune cell function, creating a profoundly immunosuppressive milieu [8,9].

This review distinguishes itself from previous comprehensive summaries [6,7,10] through several unique contributions. First, we provide an ecosystem-wide analysis of coordinated metabolic adaptations across cancer cells, cancer-associated fibroblasts (CAFs), and immune cells, rather than focusing solely on tumor cell-autonomous metabolism. Second, we introduce the metabolic-immune checkpoint as an integrated conceptual framework, proposing that programmed cell death protein 1 (PD-1), its ligand programmed death-ligand 1 (PD-L1), and other immune checkpoints function as downstream effectors of a broader metabolic surveillance system. Third, we incorporate recent evidence, including single-cell and spatial metabolomics studies revealing metastasis-specific vulnerabilities [11], lactate-driven PD-1 regulation on Tregs [12], and the influence of the gut microbiome and Helicobacter pylori (H. pylori) on the GC immune-metabolic landscape [13,14,15,16,17,18]. Finally, we provide a critical translational perspective, evaluating ongoing trials, analyzing past failures, and proposing biomarker-driven patient stratification based on metabolic subtypes [19,20]. Taken together, this review positions the metabolic-immune checkpoint as a central organizing principle for guiding personalized combination therapies in GC.

## 2 The Metabolic Landscape of the GC TME

The GC TME is a metabolically hostile niche, primarily characterized by hypoxia, acidosis, and profound nutrient deprivation. This severe milieu arises from the combination of aberrant, inefficient vasculature and the insatiable anabolic demands of rapidly proliferating tumor cells [4]. Far from being a passive backdrop, these harsh conditions impose a powerful selective pressure, driving extensive metabolic adaptations not only in the cancer cells themselves but across the entire ecosystem of resident stromal and immune cells [8].

### 2.1 Metabolic Reprogramming of GC Cells

At the core of this metabolic dysregulation are the GC cells, which undergo comprehensive rewiring of their metabolic circuitry to fulfill the biosynthetic and bioenergetic requirements of malignant growth. This reprogramming extends beyond Adenosine Triphosphate (ATP) generation to support the production of macromolecules for biomass accumulation, maintain redox homeostasis, and activate pro-proliferative signaling pathways. A cornerstone of this adaptation is the Warburg effect, or aerobic glycolysis, wherein GC cells preferentially ferment glucose to lactate even in the presence of oxygen [21]. While less efficient for ATP yield per glucose molecule compared to oxidative phosphorylation, this metabolic strategy provides a rapid ATP supply and, more importantly, shunts glycolytic intermediates (such as glucose-6-phosphate and 3-phosphoglycerate) into branching anabolic pathways for the synthesis of nucleotides, amino acids, and lipids. This glycolytic flux is driven by the upregulation of key enzymes, including hexokinase 2 (HK2), pyruvate kinase M2 (PKM2), and lactate dehydrogenase A (LDHA). HK2 overexpression is closely associated with aggressive tumor behavior and trastuzumab resistance, regulated by factors such as the stem cell factor SALL4 and circadian regulators including PER1 [6,22]. Similarly, PKM2 plays a context-dependent role in GC progression, influenced by E-cadherin expression, and drives tumor growth through mechanisms such as p65 stabilization which enhances Bcl-xL transcription and modulation of the PI3K/AKT/mTOR pathway [6,23,24]. In parallel, GC cells frequently develop a marked dependency on glutamine, supported by diverse regulatory networks including the CDC42/GLS1 [25] axis, circRNA-mediated upregulation of glutamine transporters [26], and lncRNA-driven stabilization of glutaminase mRNA [6,27].

The stabilization of hypoxia-inducible factor-1α (HIF-1α), a frequent occurrence in GC, acts as a master transcriptional regulator of this glycolytic switch and many other adaptive responses. Proteomic studies in other malignancies, such as hepatocellular carcinoma, have further validated HIF-1α-regulated metabolic enzymes as potential therapeutic targets, reinforcing the relevance of this regulatory axis in cancer metabolism [6]. Concurrently, GC cells exhibit a profound dependency on glutaminolysis. Glutamine serves as a versatile nitrogen donor and carbon source. Converted to glutamate by overexpressed glutaminase in GC and further metabolized to α-ketoglutarate (α-KG), it fulfills several critical functions, including replenishing tricarboxylic acid (TCA) cycle intermediates, supporting glutathione synthesis, and providing precursors for nucleotide biosynthesis [28].

Lipid metabolism is also fundamentally altered. GC cells upregulate *de novo* fatty acid synthesis via enzymes like ATP-citrate lyase and fatty acid synthase (FASN) to supply membrane phospholipids for new cells. Furthermore, they enhance lipid uptake through scavenger receptors like CD36 and accumulate lipid droplets, a phenotype increasingly linked to chemoresistance and metastatic potential [29]. Additionally, dysregulated metabolism of other amino acids and nucleotides is common, ensuring a steady supply of building blocks to sustain uncontrolled proliferation [30]. Recent molecular profiling has further classified GC into distinct metabolic subtypes, each with unique dependencies on glycolysis, glutaminolysis, or lipid metabolism, highlighting the need for subtype-specific therapeutic strategies [19]. Together, these interconnected metabolic alterations equip GC cells to survive, proliferate, and ultimately dominate the resource-scarce TME.

### 2.2 Metabolic Reprogramming of Stromal Cells: Focus on Cancer-Associated Fibroblasts

CAFs constitute a numerically and functionally dominant stromal population within the GC TME. Far from being inert structural components, they are metabolically dynamic and active architects of the tumor-promoting microenvironment. Their reprogramming supports tumor progression not only through physical remodeling but also via intricate metabolic crosstalk and the secretion of a potent immunosuppressive secretome [31].

A key feature of this metabolic interplay is the so-called reverse Warburg effect [32]. In this paradigm of metabolic symbiosis, cancer cells send instructive signals including reactive oxygen species (ROS), transforming growth factor-beta (TGF-β), and other cytokines that induce a state of aerobic glycolysis in neighboring CAFs. This activation drives CAFs to become high-volume producers and exporters of energy-rich metabolites, particularly lactate and pyruvate. These metabolites are subsequently imported by adjacent cancer cells via monocarboxylate transporters and funneled into their mitochondrial TCA cycle to fuel efficient oxidative phosphorylation. This metabolic coupling allows cancer cells to effectively “offshore” the glycolytic burden, optimizing their own energy production while simultaneously acidifying the shared extracellular space through CAF-derived lactic acid efflux.

The education and activation of CAFs in GC involves specific oncogenic signaling pathways. For instance, H. pylori infection can drive an NF-κB-PIEZO1-YAP1-CTGF axis that promotes CAF-mediated TME remodeling and tumor progression [33]. Beyond fueling tumor growth, CAFs exert profound immunosuppressive influence through the secretion of a diverse array of soluble factors. This immunosuppressive secretome includes chemokines like CXCL12, which recruits immunosuppressive myeloid-derived suppressor cells (MDSCs); pro-angiogenic factors such as vascular endothelial growth factor (VEGF); and cytokines including interleukin-6 (IL-6) and IL-10. These signals collectively promote the recruitment, expansion, and functional polarization of regulatory T cells (Tregs) and M2-like tumor-associated macrophages (TAMs), while directly inhibiting the activation, proliferation, and cytotoxic function of infiltrating CD8^+^ T lymphocytes. Thus, CAFs act as central metabolic and signaling hubs that shape an immunosuppressive TME, facilitating immune evasion and contributing to the failure of anti-tumor immune responses in GC.

### 2.3 Metabolic Reprogramming of Immune Cells

**Figure 1:**
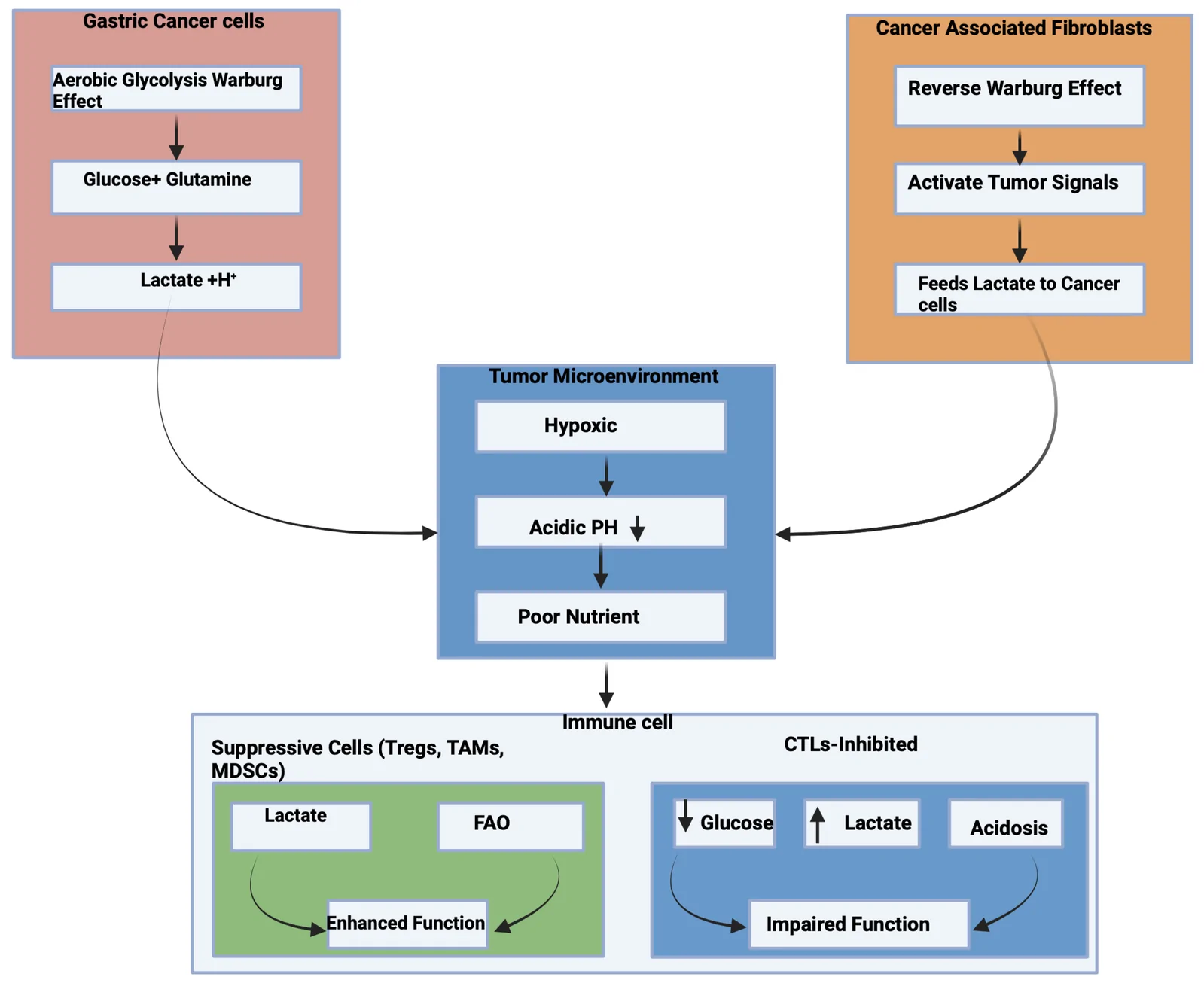
Cell-specific metabolic reprogramming in the gastric cancer (GC) tumor microenvironment (TME). The GC TME comprises multiple cell types with distinct metabolic adaptations. GC cells exhibit aerobic glycolysis (Warburg effect), consuming glucose and glutamine while producing lactate. Cancer-associated fibroblasts (CAFs) undergo the reverse Warburg effect, contributing to lactate production and metabolic coupling with tumor cells. Cytotoxic T lymphocytes (CTLs) depend on glycolysis and glutaminolysis for effector function but are inhibited by glucose competition, lactate accumulation, and acidosis. Regulatory T cells (Tregs) and M2-like TAMs preferentially utilize oxidative phosphorylation and fatty acid oxidation, thriving in the lactate-rich environment. Myeloid-derived suppressor cells (MDSCs) exhibit a hybrid glycolytic and fatty acid metabolic profile that fuels their immunosuppressive activity. Dendritic cells (DCs) are outcompeted for glutamine by cancer cells, impairing antigen presentation and T cell activation. Key therapeutic targets include metabolic enzymes, transporters, and immune checkpoints. Created with BioRender.com.

The functional potency of infiltrating immune cells is exquisitely governed by their metabolic state. The nutrient-scarce, lactate-rich, and hypoxic conditions of the GC TME create a formidable metabolic landscape that actively sculpts the immune response, ultimately promoting tolerance and immunosuppression over effective anti-tumor immunity [8] (Fig. 1). As underscored by contemporary reviews, metabolic interventions must be integrated with immunotherapy in rational combinations [34], and patient stratification based on both metabolic and immune profiles will be essential for personalized approaches [35]. This metabolic skewing manifests most detrimentally in cytotoxic T lymphocytes (CTLs), whose effector functions are critically dependent on a rapid metabolic transition to aerobic glycolysis and glutaminolysis to fuel clonal expansion, interferon-gamma (IFN-γ) production, and the biosynthesis of cytotoxic granules [36]. The GC TME systematically thwarts this requirement through a triad of metabolic assaults. First, voracious glucose consumption by tumor cells and activated stromal cells creates fierce glucose competition, starving CTLs of this essential fuel. Second, the resulting accumulation of lactate exerts direct immunosuppressive effects by inhibiting T cell signaling and function [37]. Third, the export of protons coupled with lactate efflux generates profound extracellular acidosis, which directly suppresses T cell receptor signaling. These metabolic barriers are further compounded by tumor-intrinsic alterations in pathways such as the pentose phosphate pathway and mitochondrial one-carbon metabolism, which support redox balance and nucleotide synthesis under stress [6,38,39]. Beyond these pathways, recent studies show that mitochondrial dysfunction in T cells drives exhaustion through HIF-1α-mediated glycolytic reprogramming [40], while ammonia generated through glutamine metabolism induces lysosomal and mitochondrial damage in CD8^+^ T cells [41].

In stark contrast, Tregs are metabolically equipped to prosper within this hostile milieu. They preferentially utilize Oxidative Phosphorylation (OXPHOS) and fatty acid oxidation (FAO) for energy generation pathways that remain relatively intact in the glucose-poor TME [42]. Notably, Foxp3 reprograms Treg metabolism to favor OXPHOS over glycolysis, conferring a survival advantage in low-glucose, lactate-rich environments [43]. Remarkably, lactate, which paralyzes CTLs, can actively promote Treg stability and suppressive function [44], and drives M2 macrophage polarization [45], as will be detailed in Section 3.1.

Further amplifying this suppressive network are MDSCs, whose expansion is a hallmark of GC. MDSCs exhibit a hybrid metabolic profile, engaging in both high glycolytic flux and fatty acid metabolism. These metabolic programs are not merely for energy production but are intrinsically linked to their potent immunosuppressive machinery, fueling the activity of arginase-1 (ARG1) and inducible nitric oxide synthase (iNOS) to deplete arginine, produce nitric oxide, and generate ROS, thereby effectively paralyzing T cell function [46]. Thus, the metabolic reprogramming of immune cells within the GC TME creates a self-reinforcing circuit: conditions shaped by tumor and stromal metabolism actively inhibit effector lymphocytes while selectively supporting and enhancing the function of diverse immunosuppressive populations [9]. Single-cell analyses of matched primary and metastatic lymph nodes in GC have visually captured this progression, showing a gradual depletion of cytotoxic CD8^+^ T cells and accumulation of exhausted T cells during metastatic dissemination [47]. Recent mechanistic studies reveal additional layers of this metabolic regulation. For instance, lactate accumulation in the GC TME not only inhibits CTL function but also upregulates PD-1 expression on regulatory T cells, further consolidating the immunosuppressive network [12]. Beyond lactate, other oncometabolites such as D-2-hydroxyglutarate (D-2HG) can directly impair CD8^+^ T cell metabolism and effector function, representing an additional metabolic strategy of immune evasion [48]. Furthermore, ammonia generated through glutamine metabolism in the TME induces lysosomal and mitochondrial damage in CD8^+^ T cells, leading to their dysfunction and death [41]. These findings collectively demonstrate how diverse metabolic byproducts converge to establish a multi-faceted barrier to anti-tumor immunity in GC. Importantly, this immunosuppressive circuit is dynamically shaped during disease progression. Recent single-cell RNA sequencing studies of matched primary and metastatic GC lesions reveal a progressive accumulation of exhausted CD8^+^ T alongside dynamic changes in immune checkpoint expression during metastatic dissemination, providing high-resolution evidence of how the immune landscape co-evolves with tumor metastasis [11].

### 2.4 Metabolic Supply-Demand Mismatch in the GC Tumor Microenvironment

The therapeutic potential of metabolic interventions depends critically on understanding the fundamental supply-demand imbalance within the GC TME. Different immune cell populations possess distinct metabolic preferences, yet all must function within a microenvironment characterized by glucose depletion, lactate enrichment, low pH, hypoxia, and accumulating waste products such as ammonia [9,49]. This mismatch explains why interventions that revive effector cells may inadvertently support immunosuppressive populations [9].

The GC TME is defined by profound nutrient scarcity. Cancer cells, particularly those exhibiting the Warburg effect, consume vast amounts of glucose, creating fierce competition that forces immune cells to adapt. This glucose deprivation, combined with lactate accumulation from glycolysis, fundamentally alters immune cell metabolism and function [49,50]. Additionally, hypoxia drives HIF-1α-mediated metabolic adaptations, while amino acid depletion further compromises immune responses [9,49].

#### 2.4.1 Cytotoxic T Lymphocytes

CTLs depend heavily on glycolysis and glutaminolysis to support proliferation, IFN-γ production, and cytotoxic function [51]. Upon activation, CTLs undergo a metabolic switch from OXPHOS to glycolysis, essential for providing biosynthetic precursors needed for clonal expansion. However, in the GC TME, glucose is severely depleted due to consumption by tumor cells and CAFs, while lactate accumulation directly inhibits CTL glycolysis [9,50]. This creates a metabolic crisis for CTLs, leading to impaired proliferation, reduced cytokine production, and functional exhaustion [51]. The acidic environment [9] and ammonia accumulation [41] further damage CTL function [9]. Interventions that restore glucose availability or inhibit lactate production can revive CTL function, but these same interventions may inadvertently benefit other cell types with different metabolic preferences [9].

#### 2.4.2 Regulatory T Cells

In stark contrast to CTLs, Tregs are metabolically equipped to thrive in the glucose-poor, lactate-rich TME. Tregs preferentially utilize OXPHOS and FAO for energy generation, pathways that remain intact under glucose deprivation [42]. Foxp3 reprograms Treg metabolism to favor OXPHOS over glycolysis, conferring a survival advantage in low-glucose, high-lactate environments [43]. Lactate itself promotes Treg stability and suppressive function through Foxp3 stabilization [44]. This means interventions that deplete glucose or inhibit glycolysis may paradoxically spare or enhance Treg function, potentially worsening immunosuppression. Therefore, effective metabolic immunotherapy must selectively target Treg metabolic pathways, such as FAO inhibition, while simultaneously reviving CTL glycolysis.

#### 2.4.3 Tumor-Associated Macrophages

TAMs exhibit remarkable metabolic plasticity. M1-like pro-inflammatory macrophages rely on glycolysis to support anti-tumor functions, while M2-like immunosuppressive TAMs preferentially utilize OXPHOS and FAO fueled by glutamine and fatty acids [52,53]. Lactate accumulation actively drives M2 polarization through CD36-mediated lipid uptake and PPAR-γ signaling, creating a self-reinforcing cycle [53]. Hypoxia stabilizes HIF-1α in TAMs, further promoting M2 polarization [52]. Interventions targeting FAO or glutamine metabolism may impair M2 TAM function, but may also affect other FAO-dependent populations such as Tregs, necessitating careful consideration of off-target effects [53].

#### 2.4.4 Myeloid-Derived Suppressor Cells

MDSCs exhibit a hybrid metabolic profile, engaging in both glycolysis and fatty acid metabolism to fuel their immunosuppressive machinery [9,54]. Their programs support ARG1 and iNOS activity, depleting arginine and producing nitric oxide and ROS, thereby paralyzing T cell function [46]. In GC patients, circulating and tumor-infiltrating ARG1-expressing cells are primarily immature and monocytic MDSCs [55]. MDSCs are particularly dependent on arginine metabolism; ARG1 activity consumes arginine, depleting this semi-essential amino acid from the TME and directly impairing T cell proliferation [9,54]. Their metabolic versatility makes MDSCs difficult to target, as inhibition of a single pathway may be compensated by upregulation of alternative pathways. Interventions that deplete arginine may inadvertently impair CTL function while sparing MDSCs [9].

#### 2.4.5 Dendritic Cells

DCs are critical for initiating anti-tumor immune responses through antigen presentation and T cell activation. DC activation requires a metabolic shift toward glycolysis, but DCs are highly sensitive to nutrient competition. Cancer cells outcompete DCs for glutamine via SLC38A2, impairing antigen presentation and T cell activation [56]. Glutamine deficiency reduces cDC1 numbers and frequency, with cDC1s being particularly sensitive to low glutamine levels [57]. This is mediated through reduced survival, proliferation, and maturation of cDC1s [57]. Additionally, lactate accumulation inhibits DC maturation, while adenosine signaling suppresses DC antigen presentation capacity [9]. Interventions restoring glutamine availability [57] or blocking adenosine signaling [9] could enhance DC function and improve T cell priming, but must be balanced to avoid inadvertently supporting tumor growth or Treg function [9].

#### 2.4.6 Therapeutic Implications of the Supply-Demand Mismatch

This analysis reveals critical insights for therapeutic design. First, glycolysis inhibition may impair CTL function while sparing Tregs and MDSCs, explaining why glycolysis inhibitors show limited efficacy as monotherapies [9]. Second, FAO targeting may impair Treg and M2 TAM function, but must be monitored for effects on other FAO-dependent cells [53]. Third, glutamine metabolism is a complex target; glutamine is essential for both tumor growth and DC function, requiring strategies that selectively disrupt tumor glutamine utilization while preserving immune cell function [57]. Fourth, arginine metabolism illustrates the delicate balance required: inhibiting ARG1 can restore arginine for CTLs, but must be combined with interventions addressing upstream MDSC accumulation and checkpoint upregulation [54,55]. Successful metabolic immunotherapy requires strategically rebalancing the entire metabolic ecosystem to favor effector populations while dismantling support for immunosuppressive cells [9,53].

## 3 Immunosuppressive Metabolic Pathways and Mediators

The rewired metabolism of the GC ecosystem generates a constellation of specific metabolites that function as potent soluble mediators of immune suppression. These molecules create biochemical barriers that actively restrain anti-tumor immunity while fostering a tolerant microenvironment.

### 3.1 The Lactate Axis

Once dismissed merely as a metabolic waste product of glycolysis, lactate has emerged as a central immunosuppressive metabolite and a multifunctional signaling molecule within the GC TME. LDHA-driven lactate production actively blunts tumor immunosurveillance by inhibiting the function of T cells and natural killer cells [58], creating a self-reinforcing immunosuppressive circuit. Its accumulation, primarily resulting from the Warburg effect in tumor cells and the reverse Warburg effect in CAFs, orchestrates a broad spectrum of immunomodulatory effects [45]. The immunosuppressive mechanisms of lactate are multifaceted. Its active export from glycolytic cells into the extracellular space via MCTs, particularly MCT4, is the primary driver of TME acidosis, establishing an extracellular pH often ranging from 6.0 to 6.8. This acidic milieu directly inhibits T cell receptor signaling and impairs the cytotoxicity of natural killer (NK) cells. Beyond its role in establishing acidosis, lactate itself acts as a signaling molecule. It can be imported via MCT1 into cells with active oxidative metabolism, serving as an alternative fuel source. Intracellularly, lactate inhibits histone deacetylases (HDACs), leading to hyperacetylation of histones and transcription factors, thereby altering the epigenetic landscape and gene expression profiles of immune cells [1]. Notably, in highly glycolytic TME, lactate actively promotes PD-1 expression on regulatory T cells, enhancing their immunosuppressive capacity and creating a direct link between glycolytic metabolism and immune checkpoint regulation [12]. This mechanism represents a feed-forward loop where lactate production both directly inhibits effector cells and simultaneously strengthens regulatory populations. As previously detailed, these combined effects directly cripple the proliferative capacity, cytokine production (notably IFN-γ and TNF-α), and cytotoxic function of CD8^+^ T cells and NK cells. Conversely, lactate promotes the differentiation, stability, and suppressive function of Tregs and drives the polarization of TAMs toward an M2 phenotype, effectively reinforcing the immunosuppressive network [44,45].

This pivotal role of lactate has made its production and transport attractive therapeutic targets. Strategies aim to inhibit LDHA to block lactate synthesis or to pharmacologically antagonize MCT1 and MCT4 to prevent its export and intercellular shuttling. Compounds such as AZD3965, a selective MCT1 inhibitor, have entered clinical trials based on promising preclinical data showing that disrupting lactate flux can alleviate metabolic immunosuppression and synergize with other therapies [59]. By targeting the lactate axis, these interventions seek to neutralize a key metabolic driver of immune evasion in GC. Recent evidence also shows that lactate accumulation upregulates PD-1 expression on Tregs in highly glycolytic TME, creating a direct link between metabolism and checkpoint expression [12].

### 3.2 The Adenosine Pathway

The ectonucleotidase-mediated generation and subsequent signaling of extracellular adenosine constitute one of the most potent and evolutionarily conserved pathways of immunosuppression within the TME. In GC, the dysregulated activity of this pathway establishes a powerful biochemical barrier that effectively quenches anti-tumor immune responses [60]. Adenosine is generated in the extracellular space through a tightly coupled two-step enzymatic cascade. The process initiates with the release of ATP from stressed or dying cells, a common occurrence in the necrotic core of rapidly growing tumors. This extracellular ATP is rapidly hydrolyzed to AMP by the ectoenzyme CD39 (ectonucleoside triphosphate diphosphohydrolase 1). AMP is then converted into immunosuppressive adenosine by the action of CD73 (5′-ectonucleotidase). Critically, both CD39 and CD73 are frequently overexpressed on the surface of GC cells, CAFs, and specific immune subsets notably Tregs creating a pervasive network for adenosine production that envelopes infiltrating lymphocytes [60,61].

The resultant accumulation of adenosine exerts its suppressive effects primarily by engaging high-affinity A2A and lower-affinity A2B G-protein-coupled receptors (A2AR and A2BR) on the surface of immune cells. Ligation of A2AR on CTLs and NK cells triggers adenylate cyclase activation, leading to a profound increase in intracellular cyclic AMP (cAMP) levels. This second messenger initiates a signaling cascade that suppresses T cell receptor (TCR) and cytokine receptor signaling, ultimately crippling T cell and NK cell activation, proliferation, production of effector cytokines like IFN-γ, and cytotoxic granule release. Concurrently, adenosine signaling promotes the expansion and enhances the suppressive function of Tregs and MDSCs, thereby amplifying the immunosuppressive feedback loop.

Given its central role in immune evasion, the adenosine pathway presents multiple nodes for therapeutic intervention. Current strategies aim to disrupt this axis at various levels, including CD73 inhibitors that block the final step of adenosine generation, A2A and A2B receptor antagonists that prevent adenosine from engaging its receptors on immune cells, and CD39 inhibitors that target the initiating enzyme of the cascade. These agents are frequently investigated in rational combination with ICB, such as anti-PD-1/PD-L1 therapy, with the hypothesis that relieving adenosine-mediated suppression will synergistically enhance the reinvigoration of tumor-specific T cells and improve clinical outcomes [62].

### 3.3 The Tryptophan-Kynurenine-AhR Axis

The catabolism of the essential amino acid tryptophan along the kynurenine pathway represents a critical metabolic immune checkpoint co-opted by GC to enforce local immune tolerance. In GC, indoleamine 2,3-dioxygenase 1 (IDO1) and COL12A1 form a positive feedback loop through MAPK pathway activation that promotes lymphatic metastasis [63], highlighting how tryptophan catabolism intersects with extracellular matrix remodeling to drive progression. This axis operates through a dual mechanism involving nutrient deprivation and active signaling via immunomodulatory metabolites [64]. The pathway is initiated by the rate-limiting enzymes IDO1 and, to a lesser extent, tryptophan 2,3-dioxygenase (TDO2), which catalyze the conversion of tryptophan into N-formylkynurenine, rapidly metabolized to kynurenine. IDO1 is frequently overexpressed in GC by tumor cells themselves, as well as by tumor-infiltrating dendritic cells and macrophages, establishing a robust enzymatic barrier that depletes tryptophan from the local microenvironment. This scarcity activates the general control nonderepressible 2 kinase-mediated integrated stress response in T cells, leading to cell cycle arrest and functional anergy.

More potently, the accumulating kynurenine and its downstream metabolites serve as endogenous ligands for the aryl hydrocarbon receptor (AhR), a ligand-activated transcription factor expressed on various immune cells. AhR activation orchestrates a broad immunosuppressive program: it promotes the differentiation of naïve CD4^+^ T cells into Tregs, drives TAMs toward an M2-like, pro-tumorigenic phenotype, and can directly inhibit the effector functions of CD8^+^ T cells and NK cells. Thus, this axis creates a self-amplifying loop where tryptophan catabolism both starves effector lymphocytes and generates metabolites that actively instruct immunosuppressive cell fates.

Therapeutically, targeting this axis has faced significant challenges, most notably the negative outcome of the phase III ECHO-301 trial combining the IDO1 inhibitor epacadostat with pembrolizumab in melanoma [65]. This failure has prompted critical re-evaluation of the IDO1-targeting strategy. Several factors may explain the disappointing results, including the lack of biomarker-driven patient selection, as IDO1 expression and kynurenine-to-tryptophan ratios were not used as enrollment criteria, the possibility that compensatory TDO2 activity sustained tryptophan catabolism despite IDO1 inhibition, and the potential that epacadostat achieved insufficient target inhibition in the TME. These insights have refocused efforts on rational combination therapies, dual IDO1/TDO2 inhibition to overcome enzymatic redundancy, and direct AhR antagonism. In GC, the combination of nivolumab with the IDO1 inhibitor BMS986205 showed only modest activity, with a 13% objective response rate in previously treated patients and no responses in PD-1/CTLA-4-experienced patients [66]. Crucially, ongoing efforts are directed toward biomarker-driven patient selection, such as identifying tumors with high IDO1 expression or an elevated kynurenine-to-tryptophan ratio in plasma or tumor tissue, to enrich for populations most likely to benefit from pathway inhibition.

### 3.4 Arginine Metabolism

The dysregulation of arginine metabolism constitutes another fundamental mechanism of metabolic immunosuppression in GC, primarily orchestrated by tumor-infiltrating myeloid cells. This pathway operates by creating a state of functional arginine starvation for T cells, effectively paralyzing their anti-tumor capacity [55].

The core of this immunosuppressive mechanism lies in the elevated expression of ARG1 within MDSCs and M2-like TAMs. ARG1 catalyzes the hydrolysis of L-arginine to ornithine and urea, thereby depleting this semi-essential amino acid from the local microenvironment. Arginine is indispensable for T cell biology; it is required for the expression of the CD3ζ chain of the TCR complex, proper cell cycle progression, and the synthesis of polyamines and nitric oxide. Consequently, arginine scarcity induces profound T cell dysfunction: downregulation of the CD3ζ chain impairs TCR signaling, while the inhibition of mTORC1 activity leads to G0-G1 cell cycle arrest and a failure to proliferate upon antigen encounter. Concurrently, some myeloid subsets utilize iNOS to metabolize arginine into nitric oxide, a reactive molecule that can further suppress T cell function and induce apoptosis [46].

**Figure 2:**
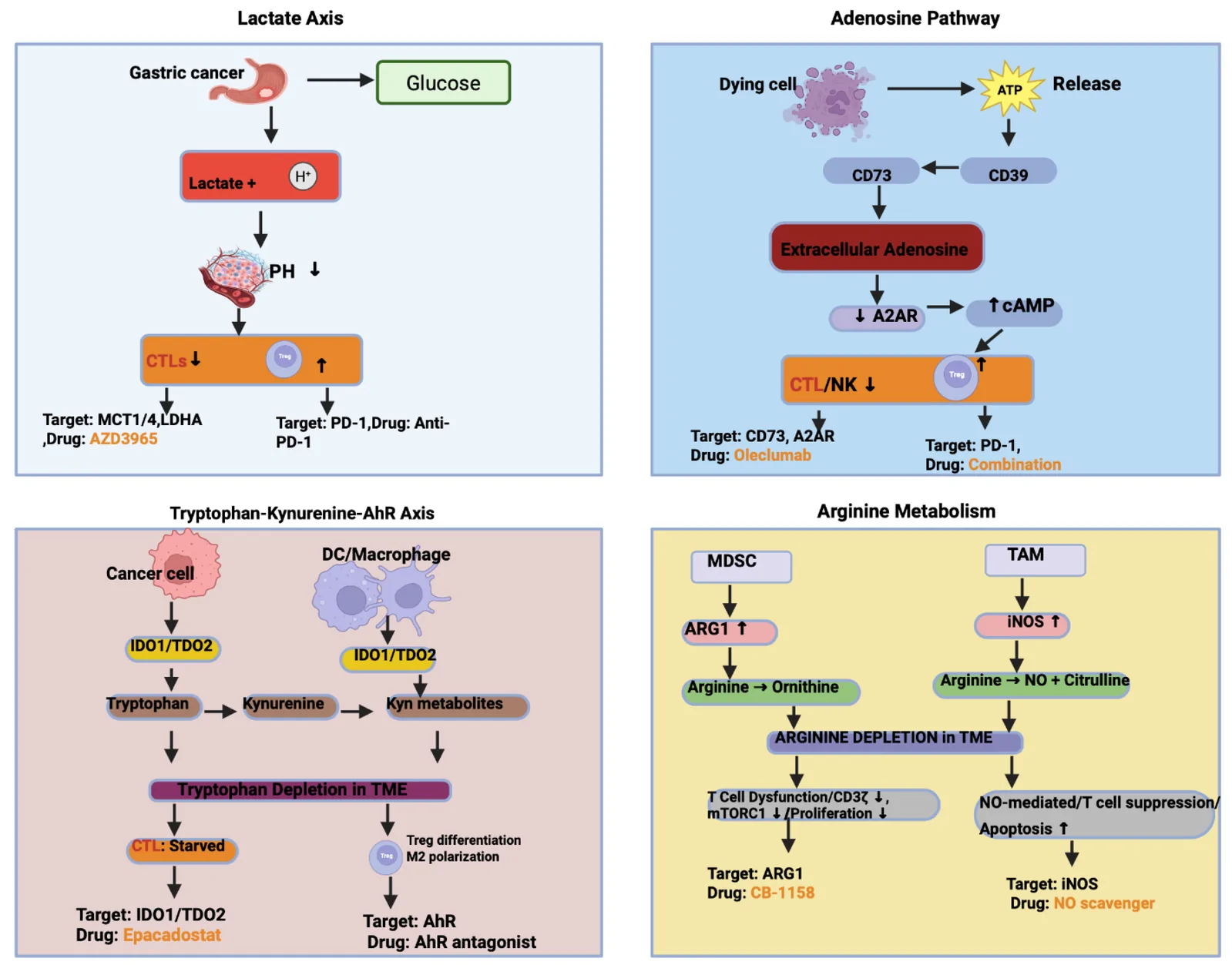
Schematic overview of the major immunosuppressive metabolic pathways operating within the GC TME. Lactate, produced by cancer cells and cancer-associated fibroblasts, promotes acidosis, inhibits cytotoxic T lymphocyte (CTL) function, drives regulatory T cell (Treg) stability, and induces M2 macrophage polarization. Adenosine, generated via the CD39/CD73 ectoenzyme cascade, suppresses CTL and natural killer (NK) cell function while promoting Treg and MDSC expansion. Kynurenine, produced by indoleamine 2,3-dioxygenase 1 (IDO1) and tryptophan 2,3-dioxygenase (TDO2)-mediated tryptophan catabolism, activates aryl hydrocarbon receptor (AhR) signaling to promote Treg differentiation and M2 macrophage polarization while inhibiting effector T cells. Arginine depletion via arginase 1 (ARG1) and inducible nitric oxide synthase (iNOS) activity in MDSCs and M2 tumor-associated macrophages (TAMs) impairs T cell proliferation and function. Dysregulated lipid metabolism supports Treg, M2 TAM, and MDSC survival and function. These interconnected metabolic pathways collectively establish a formidable biochemical barrier to anti-tumor immunity. Key enzymes, metabolites, and targeted inhibitors are highlighted. Created with BioRender.com.

The therapeutic implications of targeting this axis are conceptually straightforward but require careful implementation. Strategies include the direct pharmacological inhibition of ARG1 with agents such as CB-1158 (INCB001158), which aims to preserve intratumoral arginine levels and restore T cell function [67]. An alternative approach involves the systemic or local supplementation of arginine using arginine-loaded delivery systems to directly replenish this essential amino acid in the TME. However, a significant challenge is that rapidly proliferating cancer cells also require arginine, and simple supplementation could inadvertently fuel tumor growth. Therefore, the most promising strategies likely involve combining ARG1 inhibition with other modalities, such as ICB or therapies that selectively impair tumor cell arginine metabolism, to create a therapeutic window where T cells are selectively rescued. Together, these four major immunosuppressive metabolic pathways, lactate, adenosine, tryptophan-kynurenine, and arginine, collectively establish a formidable biochemical barrier to anti-tumor immunity in GC. Their interconnected mechanisms and corresponding therapeutic targeting strategies are summarized in Fig. 2 and detailed in Table 1, which also includes glutamine and fatty acid metabolism as additional metabolic vulnerabilities in the GC TME.

**Table 1:** Key Immunosuppressive metabolic pathways in gastric cancer (GC) and therapeutic targets.

Pathway	Enzymes	Cell Type	Mechanisms	Therapeutic Inhibitors	Clinical Stage in GC	Evidence	Reference
Lactate Axis	LDHA, MCT1/4	Cancer cells, CAFs	Induces acidosis, inhibits CTL and NK cell function, promotes Treg stability and M2 macrophage polarization	AZD3965 (MCT1i), GNE-140 (LDHAi)	Phase I/II trials (often in solid tumors including GC)	Preclinical	[59,68]
Adenosine Pathway	CD39, CD73, A2AR/A2BR	Cancer cells, CAFs, Tregs	Elevates cAMP in T and NK cells, leading to inhibition of effector function; promotes Treg and MDSC expansion	Oleclumab (Anti-CD73), Ciforadenant (A2ARi), TTX-030 (CD39i)	Phase I/II trials (combinations with ICB)	Preclinical and clinical	[69,70,71]
Tryptophan-Kynurenine	IDO1, TDO2, AhR	Cancer cells, DCs, macrophages	Depletes tryptophan and activates AhR via kynurenine, promoting Treg differentiation, M2 polarization, and T cell inhibition	Epacadostat (IDO1i), NLG919 (IDO1i), AhR antagonists	Phase II/III (post-ECHO-301, refined trials)	Preclinical and clinical	[65,72]
Arginine Metabolism	ARG1, iNOS	MDSCs, M2 TAMs	Depletes arginine causing T cell dysfunction; produces nitric oxide leading to immune suppression	CB-1158 (ARG1i), NO donors/blockers	Preclinical/Early Phase I	Preclinical	[67]
Glutamine Metabolism	Glutaminase (GLS)	Cancer cells, MDSCs	Supports tumor growth and suppressive myeloid cell function	CB-839 (Telaglenastat, GLSi)	Phase I/II trials (combinations)	Preclinical	[73]
Fatty Acid Metabolism	FASN, CPT1A	Tregs, M2 TAMs, MDSCs	Supports Treg, M2 TAM, and MDSC function; promotes tumor growth	TVB-2640 (FASNi), Etomoxir (CPT1i)	Early-phase trials	Preclinical	[74]

Notes: LDHA (Lactate Dehydrogenase A); CAFs (Cancer-Associated Fibroblasts); Tregs (Regulatory T Cells); CTL (Cytotoxic T Lymphocyte); GC (Gastric Cancer); A2AR (Adenosine A2A Receptor); A2BR (Adenosine A2B Receptor); cAMP (Cyclic Adenosine Monophosphate); NK (Natural Killer); MDSCs (Myeloid-Derived Suppressor Cells); AhR (Aryl Hydrocarbon Receptor); ICB (Immune Checkpoint Blockade); IDO1 (Indoleamine 2,3-Dioxygenase 1); TDO2 (Tryptophan 2,3-Dioxygenase); DCs (Dendritic Cells); FASN (Fatty Acid Synthase); ARG1 (Arginase 1); iNOS (Inducible Nitric Oxide Synthase); NO (Nitric Oxide); GLS (Glutaminase); CPT1A (Carnitine Palmitoyl Transferase 1A); CD (Cluster of Differentiation); M2 (M2-like macrophages); TAMs (Tumor-Associated Macrophages); TTX-030 (CD39i) (a first-in-class, fully human anti-CD39 monoclonal antibody that functions as a CD39 inhibitor); ECHO-301 (the phase III clinical trial ECHO-301/KEYNOTE-252 evaluating epacadostat plus pembrolizumab versus placebo plus pembrolizumab in unresectable or metastatic melanoma); MCT1i (Monocarboxylate Transporter 1 inhibitor); LDHAi (Lactate Dehydrogenase A inhibitor).

### 3.5 Clinical Translation, Evidence, and Challenges

Despite decades of research, the clinical translation of metabolic immunomodulation in GC remains nascent, and the strength of evidence supporting individual pathways varies considerably.

Lactate-mediated immunosuppression is among the best-supported mechanisms in GC. Recent evidence demonstrates that lactate functions as both an energy substrate and a signaling molecule, promoting TME acidification, immunosuppression, and epigenetic reprogramming, thereby reshaping GC immune evasion and therapeutic resistance [75]. Beyond its metabolic role, lactate-induced histone lactylation has emerged as a critical metabolic-epigenetic bridge that upregulates PD-L1 expression and promotes M2 macrophage polarization in gastrointestinal cancers [76]. This lactylation-macrophage interplay represents a novel therapeutic target axis [76]. However, direct experimental evidence for histone lactylation in GC remains limited. Furthermore, many mechanistic insights, particularly lactate signaling via GPR81, remain extrapolated from melanoma and breast cancer models, requiring direct validation in the GC microenvironment.

The adenosine pathway is mechanistically well-defined, with GC stem cells exhibiting elevated CD39 expression and enhanced capacity to hydrolyze ATP, leading to increased extracellular adenosine production [61]. The development of CD73 inhibitors and A2AR antagonists in GC is at an early stage, and no efficacy data from randomized trials are available.

The tryptophan-kynurenine-AhR axis exemplifies the dangers of over-reliance on preclinical models. Despite robust preclinical evidence, the phase III ECHO-301 trial failed to show benefit from IDO1 inhibition in melanoma [65]. In GC, the combination of nivolumab with IDO1 inhibitor BMS986205 showed only modest activity, with an objective response rate of 13% in previously treated patients and no responses in PD-1/CTLA-4-experienced patients [66]. This highlights the need for biomarker-driven patient selection and combination strategies.

Arginine metabolism, while a compelling mechanism in MDSC biology, has GC-specific evidence. Studies demonstrate that circulating and tumor-infiltrating ARG1-expressing cells in gastric adenocarcinoma patients are primarily immature and monocytic MDSCs [55]. Furthermore, a distinct subset of CD45^+^CD33^low^CD11b^dim^ MDSCs suppresses CD8^+^ T cell activity via the IL-6/IL-8-arginase I axis, with serum levels of IL-6, IL-8, and arginase I positively correlating with GC progression and negatively correlating with patient survival [77]. This provides strong GC-specific evidence for arginine-mediated immunosuppression.

Importantly, GC is not a single disease. The TCGA molecular classification, EBV, MSI, CIN, and GS, identifies distinct tumor biology that may influence metabolic-immune interactions [2]. EBV-positive tumors are characterized by frequent PD-L1/2 amplification, DNA hypermethylation, and dense immune infiltration. MSI-high tumors show hypermutation with increased TILs. Chromosomal instability (CIN) tumors exhibit RTK/RAS pathway activation and TP53 mutations, while genomically stable (GS) tumors show diffuse histology with CDH1 and RHOA mutations [78,79]. HER2 amplification, predominantly observed in the CIN subtype, activates PI3K/AKT/mTOR signaling, promoting glycolysis and potentially influencing both trastuzumab and immunotherapy responses [6], while H. pylori status alters both gastric metabolism and immune infiltration [13,14,15,16,17,18,80,81,82].

Collectively, these observations reveal a field rich in biological plausibility but with variable clinical translation. The path forward requires GC-specific mechanistic studies, biomarker-driven trial designs, and integration of molecular subtype information into metabolic targeting strategies. A summary of GC-specific evidence for each immunosuppressive metabolic pathway is provided in Table 2.

**Table 2:** GC-specific evidence for immunosuppressive metabolic pathways.

Pathway	Key Molecule	GC-Specific Evidence	Experimental Model	Clinical Relevance	Therapeutic Agent	References
Lactate Axis	Lactate, LDH	Elevated LDH correlates with poor prognosis and immunotherapy resistance in GC patients; lactate directly upregulates PD-1 on Tregs in GC TME	GC patient samples, *in vitro* GC cell lines	Prognostic biomarker; target for combination with ICB	AZD3965 (MCT1i), GNE-140 (LDHAi)	[12,75]
Lactate Axis	Histone lactylation	Lactate-induced histone lactylation upregulates PD-L1 and promotes M2 macrophage polarization in gastrointestinal cancers (review; evidence largely from melanoma and colorectal cancer models)	GC cell lines and mouse models	Epigenetic target for immunotherapy sensitization	Under investigation	[76]
Adenosine Pathway	CD39, CD73, A2AR	GC stem cells show elevated CD39 expression with enhanced ATP hydrolysis capacity	GC stem cell models	Poor prognosis; potential ICB combination target	Oleclumab (anti-CD73), TTX-030 (CD39i)	[61]
Tryptophan-Kynurenine	IDO1, AhR	IDO1 overexpression correlates with lymphatic metastasis in GC; combination nivolumab + IDO1i showed modest activity (13% ORR) in GC patients	GC patient samples, phase II trial	Biomarker for metastasis; modest clinical activity	Epacadostat, BMS986205	[63,66]
Arginine Metabolism	ARG1, iNOS	Circulating ARG1+ cells in GC patients are immature monocytic MDSCs; CD45^+^CD33^low^CD11b^dim^ MDSCs suppress CD8^+^ T cells via IL-6/IL-8-arginase I axis	GC patient blood and tumor samples	Prognostic biomarker; target for T cell restoration	CB-1158 (ARG1i)	[55,77]
Glutamine Metabolism	GLS, ASCT2	Glutamine dependency in GC subtypes; targeting ASCT2 or glutamine synthetase suppresses GC cell growth	GC cell lines, xenograft models	Subtype-dependent therapeutic vulnerability	CB-839 (GLSi)	[6,83]

Notes: LDH (Lactate Dehydrogenase); Tregs (Regulatory T Cells); TME (Tumor Microenvironment); GC (Gastric Cancer); ICB (Immune Checkpoint Blockade); IDO1 (Indoleamine 2,3-Dioxygenase 1); AhR (Aryl Hydrocarbon Receptor); ORR (Objective Response Rate); ARG1 (Arginase 1); ASCT2 (Alanine-Serine-Cysteine Transporter 2); GLS (Glutaminase); MDSCs (Myeloid-Derived Suppressor Cells Tregs (Regulatory T Cells); PD-L1 (Programmed Death-Ligand 1); PD-1 (Programmed Cell Death Protein 1); CD39 (Cluster of Differentiation 39); CD73 (Cluster of Differentiation 73); A2AR (Adenosine A2A Receptor); iNOS (Inducible Nitric Oxide Synthase); BMS986205 (an investigational IDO1 inhibitor).

### 3.6 Integrated Mechanistic Framework of Metabolic Immunosuppression

While the preceding sections described the lactate, adenosine, tryptophan-kynurenine, and arginine pathways individually, these metabolic axes are not independent parallel mechanisms. Rather, they function as complementary components of a unified immunosuppressive network that operates through four sequential mechanistic layers, ultimately converging on immune checkpoint upregulation and T cell dysfunction.

#### 3.6.1 Substrate Deprivation and Metabolic Stress

The first mechanistic layer operates through nutrient competition and metabolic stress, establishing the foundational barrier that T cells must overcome to mount effective anti-tumor responses. The lactate axis, driven by glycolytic tumor cells and CAFs, depletes glucose and creates an acidic microenvironment through profound lactate accumulation [75,84]. GC is a highly glycolytic tumor characterized by elevated LDHA expression and substantial lactate production, with lactate concentrations markedly elevated in the GC TME [85]. The arginine axis, mediated by MDSC-expressed ARG1, depletes arginine, an essential amino acid required for T cell proliferation and CD3ζ chain expression [86]. The tryptophan-kynurenine axis, via IDO1 activity, depletes tryptophan, activating the GCN2 stress response in T cells and inducing cell cycle arrest. The adenosine pathway, while not directly depleting a nutrient, imposes metabolic stress through cAMP-mediated suppression of mitochondrial function and T cell metabolism [87,88]. Together, these substrate-deprivation mechanisms create a metabolically hostile environment that primes T cells for dysfunction.

#### 3.6.2 Suppressed Effector Signaling Pathways

The second layer directly impairs TCR and cytokine signaling, crippling the core pathways required for T cell activation and effector function. Lactate-mediated acidosis inhibits TCR signaling through reduced ZAP70 phosphorylation and impaired Ca^2+^ flux, directly suppressing T cell proliferation and cytokine production [75]. Adenosine, generated via the CD39/CD73 ectoenzyme cascade from extracellular ATP, engages A2A and A2B receptors on T cells, triggering adenylate cyclase activation and cAMP elevation, which suppresses TCR signaling and inhibits IFN-γ production [87,88]. Arginine depletion leads to downregulation of the CD3ζ chain, a critical component of the TCR complex, directly impairing TCR signal transduction and T cell function [86]. Kynurenine, via aryl hydrocarbon receptor (AhR) activation, suppresses signal Transducer and Activator of Transcription 1 (STAT1) and STAT4 signaling, reducing IFN-γ production and promoting T cell exhaustion [89]. These disruptions converge to cripple the signaling machinery required for effective anti-tumor immunity.

#### 3.6.3 Supported Immunosuppressive Phenotypes

The third layer reinforces the immunosuppressive network by selectively supporting regulatory populations while suppressing effector cells. Lactate promotes Treg stability and suppressive function through MOESIN-mediated enhancement of TGF-β signaling and Foxp3 stabilization [75]. Lactate also promotes M2 macrophage polarization and drives Treg recruitment via GPR81-mediated CX3CL1 induction [85]. Adenosine signaling promotes Treg expansion and MDSC accumulation through A2A/A2B receptor engagement, establishing a self-amplifying immunosuppressive circuit [87,88]. Kynurenine-AhR signaling drives naïve CD4^+^ T cell differentiation toward Tregs and polarizes macrophages to an M2-like phenotype [89]. Arginine metabolism via iNOS produces nitric oxide, a key effector molecule that mediates MDSC immunosuppressive activity and directly suppresses effector T cell function [46]. This layer establishes a self-reinforcing circuit where the TME actively generates and sustains regulatory populations.

#### 3.6.4 Convergence on Immune Checkpoint Upregulation

Critically, all four metabolic axes converge on the common endpoint of immune checkpoint upregulation, establishing what we term the metabolic-immune checkpoint axis. Lactate directly upregulates PD-1 expression on Tregs in highly glycolytic TME through MCT1-mediated uptake and NFAT1 nuclear translocation [12,75]. Histone lactylation, a novel epigenetic modification driven by lactate accumulation, has emerged as a critical metabolic-epigenetic bridge that upregulates PD-L1 expression and promotes immunosuppression [75,90]. Hypoxia and metabolic stress, induced by glucose deprivation and lactate accumulation, stabilize HIF-1α, which transcriptionally upregulates PD-L1 on tumor cells and myeloid cells via direct promoter binding [84]. The adenosine pathway upregulates PD-1 expression on CD8^+^ T cells through cAMP-mediated signaling while also inducing PD-L1 expression on tumor cells via A2BR signaling [87,88]. Kynurenine-AhR activation induces PD-1 and CTLA-4 expression on T cells and promotes PD-L1 expression on tumor cells [89]. Arginine depletion and nitric oxide production have been shown to upregulate PD-L1 on MDSCs and tumor cells through STAT1 and STAT3 signaling [86,91]. This convergence explains why tumors with high activity across multiple metabolic axes show primary resistance to ICB and why metabolic modulation may be essential to overcome immunotherapy resistance.

#### 3.6.5 Therapeutic Implications and Testable Predictions

This integrated model reveals that the four metabolic axes are not redundant parallel mechanisms but complementary components of a unified immunosuppressive network, each acting through distinct yet interconnected steps that ultimately converge on immune checkpoint upregulation. This convergence has profound therapeutic implications, suggesting that metabolic targeting and ICB are synergistic approaches that should be combined based on the dominant metabolic axis in individual patients. The model generates several specific testable predictions. First, dual-axis inhibition is expected to show greater checkpoint reduction than single-axis inhibition, as targeting multiple layers of the cascade may be necessary to overcome compensatory mechanisms. Second, tumors with dominant lactate axis activity are predicted to show predominantly PD-1 upregulation on regulatory T cells, while those with dominant tryptophan-kynurenine axis activity may show PD-1 and CTLA-4 upregulation on effector T cells, reflecting axis-specific checkpoint expression patterns. Third, patients with high activity across multiple metabolic axes are predicted to exhibit primary resistance to anti-PD-1/PD-L1 therapy, as the convergent nature of the network suggests that single-agent checkpoint blockade may be insufficient when multiple immunosuppressive layers are active. Finally, comprehensive metabolic profiling of the tumor microenvironment, incorporating lactate levels, kynurenine-to-tryptophan ratios, adenosine pathway activity, and arginase expression, holds promise for stratifying patients and guiding rational combination strategies that match specific metabolic vulnerabilities with targeted interventions. These predictions provide a framework for future preclinical and clinical studies designed to validate the integrated model and translate it into personalized therapeutic approaches for GC patients.

## 4 Interplay with Immune Checkpoints: A Metabolic Perspective

Emerging evidence reveals that immune checkpoint molecules are not merely cell-surface inhibitors of receptor signaling but are integral components of a broader metabolic control system that governs T cell fate and function. Their expression, ligation, and downstream effects are deeply intertwined with cellular metabolism, creating a bidirectional relationship where metabolic stress induces checkpoint expression, and checkpoint engagement, in turn, enforces metabolic dysfunction [92]. For example, the PD-1 signaling actively reprograms T cell metabolism by suppressing glycolysis while promoting fatty acid oxidation, forcing T cells into a catabolic state incompatible with effector functions [92]. This metabolic regulation extends beyond T cells; PD-L1 expression on tumor-associated dendritic cells protects them from ferroptosis during immunogenic chemotherapy, linking checkpoint signaling to critical cell survival pathways [93].

Critically, this bidirectional crosstalk establishes what can be conceptualized as an integrated metabolic-immune checkpoint axis. In this framework, the metabolic stress imposed by the GC TME, including hypoxia, glucose deprivation, lactate accumulation, and ROS, actively drives PD-L1 upregulation on tumor cells and myeloid cells through HIF-1α stabilization, AKT/mTOR signaling, and STAT3 activation [94]. Concurrently, PD-1 engagement on T cells imposes a metabolic barrier by suppressing glycolysis and promoting fatty acid oxidation [92], effectively trapping T cells in a state of metabolic dysfunction that further reinforces exhaustion. This creates a self-perpetuating circuit in which metabolic stress drives PD-L1 upregulation, which in turn promotes PD-1 engagement and T cell metabolic suppression, leading to impaired anti-tumor immunity, sustained tumor growth, and continued metabolic stress. Understanding PD-1/PD-L1 as an integrated metabolic-immune checkpoint rather than as isolated signaling molecules has profound therapeutic implications, suggesting that metabolic modulation and ICB are not independent strategies but potentially synergistic ones.

The connection is particularly well-defined for the PD-1 pathway. Engagement of PD-1 on T cells by its ligands PD-L1 or PD-L2 transmits a potent metabolic signal that actively suppresses glycolysis, the primary energetic pathway for effector T cells while promoting a metabolic shift towards FAO. This reprogramming forces T cells from an anabolic, proliferative state into a catabolic, energy-conserving mode that is incompatible with robust effector functions, directly contributing to the hypofunctional exhausted phenotype [92]. Conversely, the TME employs metabolic sensors to upregulate PD-L1 expression as a defense mechanism. Hypoxia induces PD-L1 expression via HIF-1α stabilization [94], while oncogenic signaling pathways such as AKT/mTOR and STAT3 also transcriptionally upregulate PD-L1 [6]. Furthermore, metabolic stressors such as lactate and ROS have also been shown to increase PD-L1 expression on both tumor cells and myeloid cells, creating a feed-forward loop where a metabolically hostile TME enhances its own immune-inhibitory shield [12]. This interplay exhibits spatial and temporal dynamics during cancer progression. In GC, single-cell analyses demonstrate that immune checkpoint expression on tumor-infiltrating T cells follows a non-linear trajectory during metastasis, with initial downregulation in micro-metastatic niches potentially facilitating immune evasion, followed by upregulation in established macro-metastases, suggesting phased adaptation strategies [11]. While less studied from a metabolic standpoint, cytotoxic T-lymphocyte-associated protein 4 (CTLA-4) engagement also appears to constrain T cell glycolytic metabolism, though its dominant mechanism of action remains the competitive disruption of CD28-mediated co-stimulation.

This intricate crosstalk carries profound therapeutic implications. It suggests that ICB and metabolic modulation are not independent strategies but potentially synergistic ones. The metabolic dysfunction imposed by the TME may establish a primary layer of resistance to ICB; T cells that are metabolically starved or poisoned may be incapable of reinvigoration by PD-1/PD-L1 blockade alone. Therefore, interventions designed to reverse metabolic suppression such as alleviating glucose competition through dietary or pharmacological means, inhibiting lactate production or export, or supplementing crucial amino acids could serve to recharge or prime the T cell compartment. By restoring a metabolic profile conducive to activation and proliferation, these strategies could lower the threshold for T cell response and significantly enhance the efficacy of subsequent or concurrent ICB, offering a promising combinatorial approach to overcome resistance in GC.

### Metabolic Influences on Immunotherapy Response in GC

The metabolic landscape of the GC TME has direct implications for current immunotherapy practice, particularly in understanding response and resistance to ICB. This section integrates the metabolic-immune framework with established GC treatment contexts.

Primary resistance to PD-1/PD-L1 blockade often reflects pre-existing metabolic barriers within the TME. High glycolytic activity and lactate accumulation create an acidic, nutrient-deprived environment that impairs T cell infiltration and function, even before checkpoint engagement [6,37]. Tumors with elevated LDHA expression or MCT4-mediated lactate export may therefore be intrinsically resistant to PD-1/PD-L1 inhibition due to metabolic exhaustion of tumor-infiltrating lymphocytes [58]. Similarly, high IDO1 expression and elevated kynurenine-to-tryptophan ratios can induce T cell anergy and promote Treg differentiation, establishing a metabolic barrier that limits ICB efficacy [64,65].

Acquired resistance after initial response may arise from metabolic adaptation of tumor cells. Following PD-1/PD-L1 blockade, surviving tumor cells can upregulate compensatory metabolic pathways, such as increasing glutamine dependence or FAO, to sustain proliferation while maintaining immunosuppressive metabolite production [6]. Additionally, chronic T cell stimulation under metabolic stress promotes mitochondrial dysfunction and exhaustion through HIF-1α-mediated glycolytic reprogramming, creating a state of metabolic refractoriness to further checkpoint inhibition [40].

GC molecular subtypes exhibit differential metabolic vulnerabilities that may influence ICB response [19]. MSI-high tumors, characterized by high mutation burden and dense immune infiltration, generally show favorable responses to PD-1 blockade [78]. However, the metabolic demands of activated T cells in these tumors may be constrained by glucose competition and lactate accumulation, particularly in large, necrotic lesions [37]. EBV-positive tumors, which show frequent PD-L1 amplification and robust immune infiltration, may benefit from metabolic modulation targeting the adenosine pathway, as elevated CD39 expression on GC stem cells contributes to immunosuppressive adenosine accumulation [61]. CIN tumors, the most common subtype, often exhibit RTK/RAS pathway activation and enhanced glycolysis, potentially rendering them more susceptible to glycolysis inhibitors combined with ICB [78,79]. GS tumors, with diffuse histology and poor prognosis, may rely more heavily on lipid metabolism and FAO, suggesting that FAO inhibitors could synergize with immunotherapy in this subtype [19,79]. HER2-positive GC, predominantly within the CIN subtype, activates PI3K/AKT/mTOR signaling, promoting glycolysis and potentially influencing both trastuzumab and immunotherapy responses [6].

Potential biomarker combinations could improve patient stratification. Beyond PD-L1 combined positive score, incorporating metabolic biomarkers such as serum LDH, tumoral LDHA expression, CD73 expression, IDO1 expression, or kynurenine-to-tryptophan ratios may identify patients most likely to benefit from combined metabolic-immune strategies [12,63,75]. Metabolomic signatures derived from spatial metabolomics or serum profiling could further refine patient selection, distinguishing those with metabolically “hot” TMEs amenable to metabolic modulation from those with metabolically “cold” TMEs requiring alternative approaches [20]. Similarly, the combination of tumor mutational burden with metabolic biomarkers may identify MSI-high or EBV-positive patients who would benefit from adjunctive metabolic targeting.

Chemotherapy-immunotherapy combinations represent another context where metabolic-immune crosstalk is clinically relevant. Cytotoxic chemotherapy induces immunogenic cell death, releasing ATP that can be converted to immunosuppressive adenosine by CD39/CD73 [62]. Concurrent inhibition of the adenosine pathway may therefore enhance the immunostimulatory effects of chemotherapy and improve outcomes when combined with ICB. Similarly, chemotherapy-induced metabolic stress can upregulate PD-L1 expression on tumor cells via HIF-1α stabilization, providing a rationale for combining chemotherapy with PD-1/PD-L1 blockade [94]. Collectively, these observations suggest that metabolic profiling, integrated with established biomarkers, could guide personalized immunotherapy strategies in GC and help overcome resistance mechanisms.

## 5 Therapeutic Strategies Targeting Metabolic-Immune Crosstalk

The delineation of metabolic pathways as key regulators of immunosuppression has opened a new frontier for cancer therapy. The therapeutic goal is twofold, aiming to dismantle the tumor immunosuppressive metabolic architecture and to actively re-energize faltering anti-tumor immunity. This can be achieved through a multi-pronged approach targeting different layers of the metabolic network. One foundational strategy involves targeting tumor cell-intrinsic metabolism. Inhibiting the core metabolic engines of cancer cells such as glycolysis with agents like 2-DG or HK2 inhibitors, glutaminolysis with glutaminase inhibitors, or *de novo* lipogenesis with FASN inhibitors serves a dual purpose. For instance, targeting the glutamine transporter ASCT2 or glutamine synthetase has shown promise in suppressing GC cell growth in a subtype-dependent manner [83].  It directly impairs tumor proliferation and survival while crucially reducing the tumor output of immunosuppressive metabolites such as lactate, thereby improving the fitness of neighboring immune cells. Kynurenine, produced through tryptophan catabolism via IDO1/TDO2 rather than core metabolic pathways, represents a distinct but complementary immunosuppressive axis that can be targeted separately to relieve immune suppression [64,65].

A more direct approach is to target the immunosuppressive metabolites themselves or their signaling axes. As detailed in Table 1, this includes inhibiting LDHA or MCTs to block lactate flux, using anti-CD73/A2AR antagonists to disrupt adenosine signaling, and employing IDO1/TDO2 or AhR inhibitors to neutralize the tryptophan-kynurenine pathway. These agents aim to lift the specific biochemical brakes placed on the immune system within the TME.

A complementary and innovative strategy seeks to actively reprogram the metabolism of immune cells to favor effector over suppressive functions. Modulating cholesterol metabolism in CD8^+^ T cells by inhibiting ACAT1 has been shown to enhance their antitumor response by improving immune synapse formation [95], suggesting that lipid metabolic interventions can potentiate T cell function in the TME. This involves pharmacologically forcing a metabolic switch in favor of anti-tumor immunity. Examples include using cytokines like IL-2 or IL-12 to promote aerobic glycolysis in tumor-infiltrating lymphocytes, employing peroxisome proliferator-activated receptor alpha (PPARα) agonists to enhance FAO and modulate Treg function, or utilizing selective phosphoinositide 3-kinase delta (PI3Kδ) inhibitors which have been shown to preferentially disrupt Treg metabolism while sparing or even enhancing cytotoxic T cell responses [96,97]. The identification of metastasis-specific metabolic vulnerabilities, such as the upregulation of the mitochondrial ATP synthase subunit ATP5MC2 in early metastatic GC cells, opens new avenues for targeted intervention. Preclinical evidence suggests that ATP5MC2-driven oxidative phosphorylation reprogramming facilitates metastasis, positioning this pathway as a potential target for anti-metastatic therapies [11].

Ultimately, the greatest therapeutic promise lies in rational combination therapies. This integrative approach is supported by recent work emphasizing that understanding and targeting the metabolic interplay between GC cells and immune cells can enhance anti-tumor immune responses and provide new directions for GC treatment [9,10]. Preclinical studies in GC models demonstrate that combining glycolysis inhibitors with ICB can enhance antitumor immunity by reducing lactate secretion and T cell exhaustion [6]. Similarly, FAO inhibitors have been shown to synergize with low-dose chemotherapy to promote T-cell-dependent tumor control by targeting immunosuppressive MDSCs [98]. Metabolic modulators are unlikely to be curative as monotherapies but can be powerful partners for existing standards of care. Their combination with immunotherapy is particularly compelling; by alleviating metabolic suppression, these agents can convert an immunologically “cold” TME into a more permissive “hot” one, potentially overcoming primary or acquired resistance to ICB or adoptive cell therapies like CAR-T.

This therapeutic synergy is best understood through the integrated metabolic-immune checkpoint framework. Metabolic modulators are hypothesized to restore a metabolic state conducive to T cell activation and proliferation, thereby lowering the threshold for T cell response and enhancing the efficacy of subsequent or concurrent ICB. Rather than viewing metabolism and immune checkpoints as independent targets, this integrated perspective positions metabolic reprogramming as a prerequisite for effective ICB, addressing the fundamental barrier that metabolically dysfunctional T cells may be incapable of reinvigoration by PD-1/PD-L1 inhibition alone.

**Figure 3:**
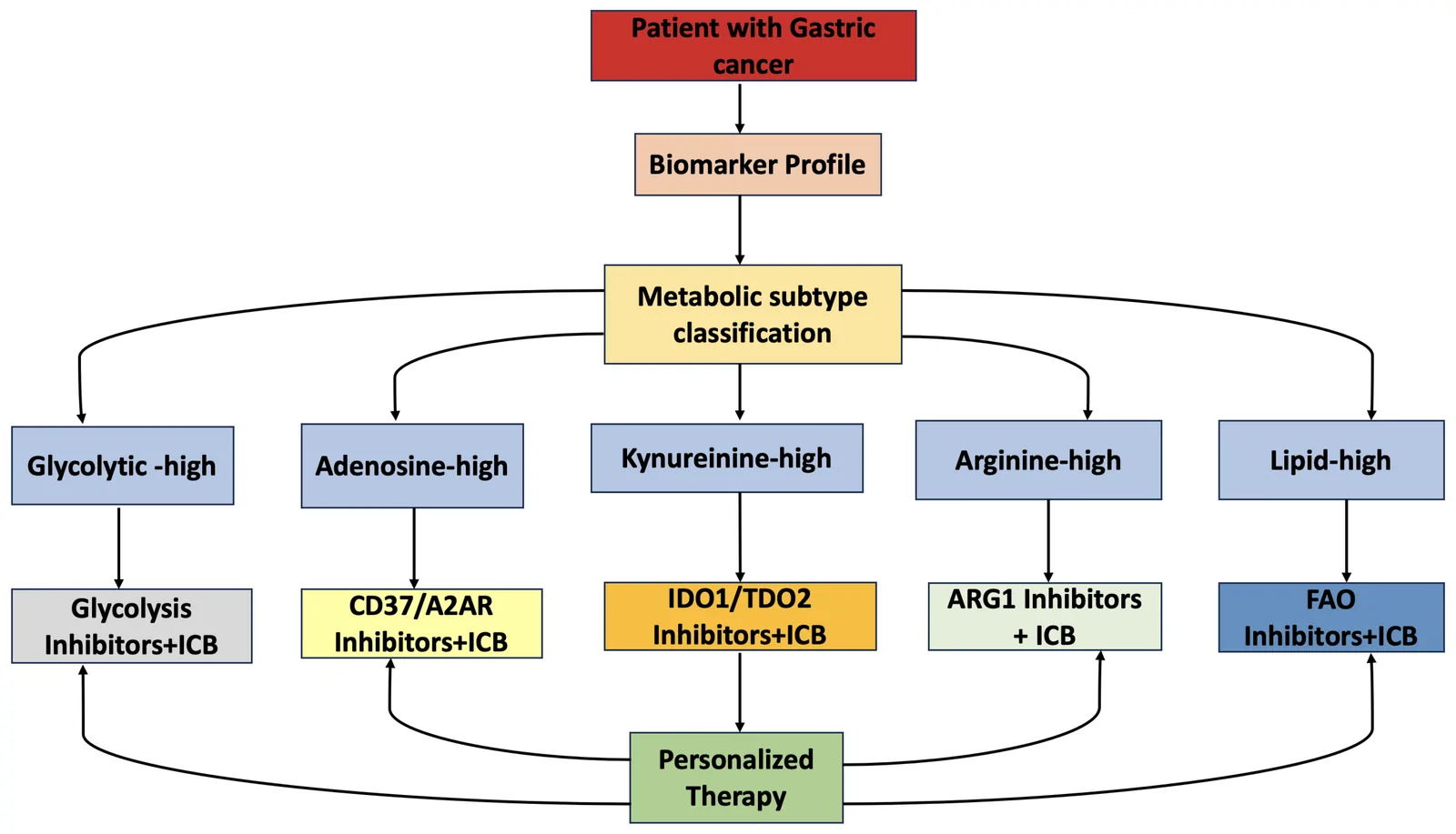
Decision framework for targeting metabolic-immune crosstalk in GC. This framework integrates biomarkers, metabolic subtypes, and personalized combination therapies for GC. Biomarker profiling includes PD-L1 CPS, tumor mutational burden, serum LDH, tumoral LDHA expression, CD73 expression, IDO1 expression, and kynurenine-to-tryptophan ratios. Based on biomarker profiles, patients are stratified into five metabolic subtypes: glycolytic-high (CIN tumors, HER2-positive), adenosine-high (EBV-positive), kynurenine-high (IDO1-positive), arginine-high (MDSC-enriched), and lipid metabolism-high (GS tumors). Corresponding therapeutic strategies include glycolysis inhibitors combined with ICB, CD73/A2AR inhibitors combined with ICB, IDO1/TDO2 inhibitors combined with ICB, ARG1 inhibitors combined with ICB, and FAO inhibitors combined with ICB. This framework guides biomarker-driven patient stratification and rational combination therapy selection. Created with Microsoft PowerPoint.

Recent advances highlight novel combination approaches. Targeting glutamine metabolism may disrupt not only tumor growth but also critical immune functions, as cancer cells outcompete dendritic cells for glutamine via SLC38A2, impairing antigen presentation and T cell activation [56]. Similarly, epigenetic regulators of metabolism, such as the m6A reader YTHDF1, represent promising targets, with siRNA-mediated inhibition shown to boost antitumor immunity in preclinical models [6,99]. These approaches, combined with ICB, may address multiple layers of metabolic immunosuppression simultaneously. Similarly, pairing metabolic inhibitors with chemotherapy or radiotherapy is rational. These cytotoxic modalities often induce immunogenic cell death, releasing tumor antigens and large amounts of extracellular ATP. Concurrently blocking the CD39/CD73/adenosine axis can prevent the conversion of this immunogenic ATP into immunosuppressive adenosine, thereby enhancing the immunostimulatory abscopal effect of treatment [62]. Multi-pathway metabolic targeting, for instance, co-inhibiting both the lactate and adenosine axes, may be necessary to achieve a durable reversal of the immunosuppressive TME and unlock robust anti-tumor immunity in GC. Beyond these immunometabolic axes, targeting core tumor metabolic enzymes presents a complementary strategy. For example, inhibiting HK2 or pyruvate kinase M2 (PKM2) can reduce glycolytic flux and lactate production, while inhibitors of carnitine palmitoyl transferase 1A disrupt fatty acid oxidation, a pathway implicated in chemoresistance and stemness [6,100]. Combining these with immunotherapies may simultaneously impair tumor growth and alleviate metabolic immunosuppression [7,74,100]. A summary of therapeutic agents targeting metabolic-immune crosstalk, including their development stage, cancer types tested, GC-specific data, and key limitations, is provided in Table 3, and a therapeutic decision framework integrating biomarkers, metabolic subtypes, and personalized combination therapies is illustrated in Fig. 3.

**Table 3:** Therapeutic agents targeting metabolic-immune crosstalk in GC.

Target/Pathway	Agent	Development Stage	Cancer Type Tested	GC-Specific Data	Combination Strategy	Key Limitation	References
Glycolysis	2-DG, HK2 inhibitors	Preclinical	Various solid tumors	Limited	ICB, chemotherapy	Off-target toxicity; metabolic compensation	[6,7]
LDHA	GNE-140	Preclinical	Various solid tumors	No	ICB	Metabolic plasticity; compensatory pathways	[68]
MCT1/4	AZD3965	Phase I/II	Solid tumors, lymphoma	Limited	ICB	Isoform redundancy; on-target toxicity	[59,68]
Glutaminase (GLS)	CB-839 (Telaglenastat)	Phase I/II	Breast cancer, RCC, solid tumors	Preclinical	ICB, chemotherapy	Subtype-dependent efficacy	[73]
FASN	TVB-2640	Phase I/II	Solid tumors	No	Taxanes	Limited GC data; toxicity concerns	[74]
CD73	Oleclumab	Phase I/II	Solid tumors (NSCLC, CRC)	No	ICB	Adenosine pathway redundancy	[69,70,71]
A2AR	Ciforadenant, AZD4635	Phase I/II	RCC, prostate, solid tumors	No	ICB	Modest activity in biomarker-selected populations	[69]
CD39	TTX-030	Phase I/II	Solid tumors	No	ICB	Limited clinical data in GC; no efficacy results reported from randomized trials	[62]
IDO1	Epacadostat	Phase III (failed)	Melanoma	No	ICB (pembrolizumab)	No biomarker selection; pathway redundancy; ECHO-301 failure	[65]
IDO1	BMS986205	Phase II	GC, other solid tumors	Yes (13% ORR)	Nivolumab	Modest activity; no responses in PD-1/CTLA-4-experienced patients	[66]
IDO1/TDO2	Navoximod (GDC-0919)	Phase I	Solid tumors	No	ICB, chemotherapy	Early stage; combination trials ongoing	[72]
AhR	AhR antagonists	Preclinical and clinical	Various cancers	No	ICB	Preclinical only	[64]
ARG1	CB-1158 (INCB001158)	Phase I/II	Solid tumors	Preclinical	ICB	Limited GC data	[67]

Notes: 2-DG (2-Deoxy-D-Glucose); A2AR (Adenosine A2A Receptor); AhR (Aryl Hydrocarbon Receptor); ARG1 (Arginase 1); CRC (Colorectal Cancer); FASN (Fatty Acid Synthase); GC (Gastric Cancer); GLS (Glutaminase); HK2 (Hexokinase 2); ICB (Immune Checkpoint Blockade); IDO1 (Indoleamine 2,3-Dioxygenase 1); LDHA (Lactate Dehydrogenase A); MCTs (Monocarboxylate Transporters); NSCLC (Non-Small Cell Lung Cancer); ORR (Objective Response Rate); RCC (Renal Cell Carcinoma); TDO2 (Tryptophan 2,3-Dioxygenase).

## 6 Challenges and Future Perspectives

The translation of metabolic immunomodulation from a compelling biological concept into effective clinical therapies for GC is fraught with significant challenges that must be systematically addressed.

### 6.1 Tumor Heterogeneity and Metabolic Plasticity

The translation of metabolic immunomodulation from a compelling biological concept into effective clinical therapies for GC is fraught with significant challenges that must be systematically addressed. A primary obstacle is the profound tumor heterogeneity and metabolic plasticity inherent to GC and its microenvironment. Cancer cells possess redundant and adaptable metabolic networks; inhibiting a single pathway, such as glycolysis, may simply trigger compensatory upregulation of glutaminolysis or fatty acid oxidation, allowing the tumor to maintain its growth and immunosuppressive output. Epigenetic regulation adds another layer of complexity to metabolic plasticity. RNA methylation pathways, particularly those involving METTL3 and YTHDF1, have been shown to regulate chemokine expression and antitumor immunity in gastrointestinal cancers [99,101]. Targeting these epigenetic-metabolic intersections may overcome compensatory adaptations and provide more durable therapeutic responses. Additionally, cellular stress pathways such as PERK-mediated ER stress serve as metabolic hubs that drive immunosuppressive macrophage polarization, suggesting that stress response inhibitors could disrupt key nodes in the metabolic network [102].

### 6.2 Spatial and Temporal Heterogeneity in Metastasis

This plasticity is further complicated by spatial heterogeneity, where metabolic dependencies may differ between the invasive margin and the hypoxic core. Recent advances in spatial metabolomics have identified distinct metabolic subtypes within GC tissues. For instance, tumor-specific and stroma-specific metabolic signatures have been linked to differential prognosis, with one subtype showing elevated nucleotide metabolism and favorable outcomes [6]. Integrating such spatial metabolic data with immune profiling could guide more precise combination therapies. Recent single-cell studies highlight that metabolic programs also vary significantly between primary tumors and metastatic lesions, with specific adaptations such as ATP5MC2-mediated oxidative phosphorylation enhancement characterizing early metastatic cells [11]. These metabolic differences between primary and metastatic lesions have critical therapeutic implications. Metastatic cancer cells often exhibit distinct metabolic dependencies that differ from their primary tumor counterparts, reflecting the unique challenges of the metastatic niche, including altered nutrient availability, oxygen tension, and extracellular matrix composition [103]. Beyond ATP5MC2-mediated oxidative phosphorylation, other metabolic adaptations have been identified in metastatic GC cells, including enhanced lipid metabolism, increased glutamine dependence, and upregulation of antioxidant pathways to withstand oxidative stress during dissemination [6,104]. Understanding these metastasis-specific metabolic vulnerabilities is essential because therapies targeting primary tumor metabolism may be ineffective against metastatic lesions, and conversely, metastasis-specific metabolic pathways may represent actionable targets for preventing or treating metastatic spread. Future studies should systematically compare the metabolic profiles of matched primary and metastatic GC specimens using spatial metabolomics and single-cell approaches to identify stage-specific metabolic dependencies and guide the development of metastasis-selective therapeutic strategies [20,105]. Metastasis remains the principal cause of GC mortality, comprising a cascade of steps from local invasion to distant colonization, each presenting therapeutic opportunities and challenges [106].

### 6.3 Advanced Analytical Tools for Metabolic and Immune Mapping

Overcoming this requires advanced analytical tools. The application of spatially-resolved metabolomics, particularly mass spectrometry imaging, on human GC specimens is essential to map the precise distribution of metabolites and enzymatic activities across the TME landscape [20]. Complementary spatial transcriptomic approaches, as applied to metastatic colorectal cancer, offer a blueprint for simultaneously mapping cellular heterogeneity, metabolic states, and spatial organization within GC metastatic niches [107]. This will identify dominant metabolic vulnerabilities in specific tumor regions and patient subsets, guiding more precise therapeutic targeting [105,108]. A summary of advanced omics technologies for mapping metabolic and immune niches in GC, including what each technology measures, what it has revealed in GC, how it can guide therapy selection, and technical limitations, is provided in Table 4.

**Table 4:** Advanced omics technologies for mapping metabolic and immune niches in GC.

Technology	What It Measures	What It Has Revealed in GC	How It Can Guide Therapy Selection	Technical Limitations	References
Spatial Metabolomics	Tissue distribution of metabolites, lipids, and metabolic intermediates at high spatial resolution	Identified distinct metabolic subtypes in GC tissues; tumor-specific and stroma-specific metabolic signatures linked to differential prognosis	Identifies region-specific metabolic vulnerabilities across tumor and stromal compartments. Guides selection of targeted metabolic inhibitors based on local metabolic dependencies.	Limited coverage of metabolites; requires fresh-frozen tissues; data interpretation challenges; low throughput	[20,105]
Spatial Transcriptomics	Gene expression profiles mapped to tissue architecture with spatial context	Revealed cellular heterogeneity and spatial organization within GC metastatic niches; identified cell-specific metabolic remodeling	Maps expression of metabolic enzymes and immune markers across tumor regions. Identifies immune-excluded versus immune-infiltrated areas to guide combination therapy strategies.	Resolution limitations; probe-based or sequencing-based trade-offs; high cost; complex data analysis	[107]
Single-Cell RNA Sequencing (scRNA-seq)	Transcriptomic profiles of individual cells, enabling cell-type identification and state characterization	Revealed progressive accumulation of exhausted CD8^+^ T cells and dynamic immune checkpoint expression during metastasis; identified distinct T cell states and metabolic heterogeneity across immune populations	Characterizes metabolic states of individual T cells to distinguish responsive versus refractory populations. Identifies cell-type-specific metabolic vulnerabilities for precision targeting.	Loss of spatial context; limited detection of low-abundance transcripts; high cost; requires dissociation of tissues	[11,48].
Multiplex Imaging	Simultaneous detection of multiple proteins (immune markers, metabolic enzymes) within intact tissue sections	Visualized gradual depletion of cytotoxic CD8^+^ T cells and accumulation of exhausted T cells in metastatic lymph nodes	Identifies metabolic niches (hypoxic cores, invasive margins, immune-excluded regions). Enables spatially-resolved biomarker analysis.	Limited multiplexing capacity (typically 10–60 markers); requires specialized equipment; data analysis complexity; can be time-consuming	[47]
Spatial Multi-Omics Integration	Combined analysis of metabolomic, transcriptomic, and proteomic data within the same spatial context	Integrated analysis of tumor heterogeneity, metabolism, and immune landscape; identified ATP5MC2-mediated OXPHOS reprogramming in early metastatic cells	Comprehensive identification of dominant metabolic vulnerabilities and immune interactions. Enables multi-modal biomarker discovery.	Technical challenges in data integration; requiring computational expertise; limited availability of matched datasets; high cost and technical complexity	[11,105]

Notes: ATP5MC2 (ATP Synthase Membrane Subunit C Locus 2); CD (Cluster of Differentiation); GC (Gastric Cancer); OXPHOS (Oxidative Phosphorylation).

### 6.4 On-Target, off-Tumor Toxicity and Drug Delivery

A second major hurdle is the risk of on-target, off-tumor toxicity. Core metabolic pathways like glycolysis, oxidative phosphorylation, and nucleotide synthesis are fundamental to healthy cells, especially those in the gut, bone marrow, and immune system. Systemic inhibition can lead to unacceptable toxicities, as seen with some broad-spectrum metabolic drugs [6].

The challenge is exemplified by glycolysis inhibitors (HK2, PKM2) which affect normal tissues with high glycolytic demands [6,109], FASN inhibitors like TVB-2640 with limited GC data and toxicity concerns [74], glutaminase inhibitors such as CB-839 which may impair lymphocyte function and intestinal homeostasis [110]. LDHA inhibition, while reducing lactate production, may affect normal glycolytic tissues [6]. CD73 inhibitors and A2A receptor antagonists may disrupt cardiovascular and immune homeostasis, as these pathways are widely expressed in normal tissues [60,62]. Similarly, IDO1 inhibitors may affect normal tryptophan metabolism essential for immune and neuroprotection [64].

The future lies in engineering tumor-selective delivery systems, including nanoparticle carriers, antibody-drug conjugates, and prodrugs activated by the tumor microenvironment. Spatially-resolved metabolomics and spatial transcriptomics could guide the design of more selective strategies by mapping regional metabolic vulnerabilities [20,105,107].

### 6.5 Predictive Biomarkers for Patient Stratification

Progress is also stymied by a critical lack of predictive biomarkers, as it is unlikely that all GC patients will benefit from any single metabolic intervention. The failure of the phase III ECHO-301 trial, which combined the IDO1 inhibitor epacadostat with pembrolizumab in melanoma without biomarker-driven patient selection, underscores the importance of stratifying patients based on metabolic vulnerabilities [65]. In GC, the combination of nivolumab with the IDO1 inhibitor BMS986205 showed only modest activity, with a 13% objective response rate in previously treated patients and no responses in PD-1/CTLA-4-experienced patients, highlighting the urgent need for biomarkers such as IDO1 expression or elevated kynurenine-to-tryptophan ratios to identify patients most likely to benefit [66]. Promising biomarker candidates include metabolic enzyme expression, as elevated LDHA and serum lactate dehydrogenase have been correlated with poor prognosis and immunotherapy resistance in GC patients [75], IDO1 overexpression correlates with lymphatic metastasis [63], CD79 expression on GC stem cells identifies patients who may benefit from adenosine-targeting strategies [61], and ARG1 expression on circulating and tumor-infiltrating MDSCs correlates with GC progression and poor survival [55,77]. Metabolomic signatures derived from spatial metabolomics could further refine patient selection by distinguishing those with metabolically “hot” TMEs amenable to metabolic modulation from those with metabolically “cold” TMEs requiring alternative approaches [20,105]. The TCGA molecular classification, EBV, MSI, CIN, and GS identifies distinct tumor biology that may influence metabolic-immune interactions, with HER2 amplification in CIN tumors activating PI3K/AKT/mTOR signaling and promoting glycolysis, potentially influencing both trastuzumab and immunotherapy responses [6,78,79]. Integrating PD-L1 combined positive score, tumor mutational burden, and metabolic biomarkers could improve patient stratification for combined metabolic-immune strategies [12,63,75].

### 6.6 The Role of the Gut Microbiome and H. pylori

Beyond cellular metabolism within the TME, emerging evidence points to the significant role of the gut microbiota in shaping the metabolic and immune landscape of GC. The gut microbiome composition has been linked to response to ICB in GC, with specific bacterial taxa associated with improved outcomes to nivolumab treatment [18]. Additionally, H. pylori infection alters miR-140 expression which in turn regulates PD-L1, linking gastric microbiology to checkpoint regulation [14]. The intestinal flora can influence local metabolite availability, systemic inflammation, and even the efficacy of immunotherapy. Recent analyses suggest that modulating the microbiome may represent a novel adjunct to metabolic and immunotherapeutic strategies, offering another layer of complexity and opportunity in personalized GC treatment [9,10]. Future studies integrating metabolomic, microbiome, and immunophenotypic data will be crucial to unravel these interactions.

The influence of gut microbiota and H. pylori on the GC immune-metabolic environment warrants dedicated attention for several reasons. First, H. pylori infection, the most significant risk factor for gastric carcinogenesis, directly alters gastric epithelial metabolism through multiple mechanisms. H. pylori virulence factors such as CagA and VacA modulate host metabolic pathways including glycolysis, lipid metabolism, and amino acid utilization, while simultaneously reshaping the local immune landscape by recruiting MDSCs and polarizing TAMs toward an immunosuppressive M2 phenotype [15]. Chronic H. pylori infection drives a metabolic shift in gastric epithelial cells toward aerobic glycolysis, creating a lactate-rich microenvironment that promotes immune evasion and facilitates malignant transformation [16]. Second, the broader gut microbiome produces a diverse array of metabolites that influence systemic and local immune-metabolic states. Short-chain fatty acids such as butyrate, propionate, and acetate, generated through bacterial fermentation of dietary fiber, have been shown to modulate T cell differentiation, promote Treg function, and influence PD-L1 expression on tumor cells [80]. Conversely, gut dysbiosis can lead to the production of pro-inflammatory metabolites such as lipopolysaccharides and secondary bile acids, which activate NF-κB signaling and promote chronic inflammation, thereby contributing to gastric carcinogenesis and metabolic reprogramming [15]. Third, the composition of the gut microbiome has emerged as a predictive biomarker for immunotherapy response in GC. Studies have identified specific bacterial taxa, including Akkermansia muciniphila, Bifidobacterium species, and Faecalibacterium prausnitzii, that are enriched in patients responding to PD-1/PD-L1 blockade, likely through their effects on systemic immune tone and metabolite production [13,18]. The mechanisms underlying this association include microbiome-derived metabolites that enhance CD8^+^ T cell infiltration, modulate dendritic cell function, and regulate the kynurenine-tryptophan axis [18]. Fourth, H. pylori status may influence the efficacy of metabolic and immunotherapeutic interventions. Eradication of H. pylori has been shown to alter the gastric metabolome and immune landscape, potentially creating a more permissive environment for immunotherapy [17]. However, the optimal timing and approach for H. pylori modulation in the context of GC therapy remain poorly defined. Collectively, these observations underscore the need for integrated approaches that consider the microbiome as a critical modulator of the GC immune-metabolic microenvironment. Future research should focus on elucidating the mechanistic links between specific microbial taxa and their metabolic products in immune checkpoint regulation, identifying microbiome-derived biomarkers that predict response to combined metabolic-immune therapies, and developing microbiome-modulating strategies, including probiotics, prebiotics, and fecal microbiota transplantation, as adjuncts to conventional GC treatments [82]. Such approaches, integrated with metabolomic and immunophenotypic profiling, hold promise for personalized therapeutic strategies in GC.

### 6.7 Optimizing Combination Regimens

Future therapeutic success will require an integrated approach that targets not only classic immunosuppressive pathways but also the upstream metabolic drivers in cancer cells that fuel the hostile TME, as part of a multi-layered strategy to restore anti-tumor immunity [6]. Crucially, this integration must be guided by a conceptual framework that treats the metabolic-immune checkpoint as an indivisible functional unit. In this paradigm, PD-1/PD-L1 and other immune checkpoints are understood not as isolated cell-surface inhibitors but as downstream effectors of a broader metabolic surveillance system that governs T cell fate. Therapeutic success will therefore depend on simultaneously addressing both sides of this axis, relieving metabolic suppression to create T cells capable of responding and blocking immune checkpoints to unleash their full effector potential. Moving beyond metabolism as a cancer cell-autonomous trait, toward recognizing it as an ecosystem-wide regulator of immunity, opens a paradigm-shifting frontier for therapy. Realizing this vision will require the rational combination of metabolic modulators with immunotherapies [34], guided by patient stratification based on integrated metabolic and immune profiling [35]. In conclusion, remodeling the metabolic landscape of GC represents a paradigm-shifting therapeutic frontier. By moving beyond viewing metabolism as a cancer cell-autonomous hallmark to understanding it as an ecosystem-wide regulator of immunity, we can design next-generation, personalized combination therapies. The clear goal is to strategically rewire the metabolic circuitry of the TME, dismantling immunosuppressive networks while re-energizing effector immune responses, thereby forging a path toward more robust and durable clinical outcomes for patients with GC.

## 7 Conclusion

Metabolic reprogramming has been unveiled as a fundamental biological orchestrator, critically shaping the immunosuppressive landscape that characterizes GC progression and therapy resistance. This review has detailed a dynamic and interconnected network where metabolism transcends mere energy production to become a language of cellular interaction within the TME. Through fierce nutrient competition, symbiotic metabolite exchange, and the secretion of soluble immunosuppressive mediators, cancer cells, CAFs, and various immune populations engage in a continuous dialogue that ultimately subverts anti-tumor immunity. Deciphering this complex metabolic crosstalk has illuminated a plethora of novel therapeutic vulnerabilities beyond traditional oncogenic drivers. The key immunosuppressive axes centered on lactate, adenosine, and tryptophan/kynurenine metabolism represent actionable targets to disrupt the self-reinforcing cycle of immune suppression. While targeting these pathways alone shows preclinical promise, their greatest potential likely resides in rational combination strategies with established modalities like ICB. By alleviating the metabolic barriers that induce T cell exhaustion and dysfunction, metabolic modulators can convert an immunologically “cold” TME into a more permissive one, thereby overcoming primary and acquired resistance to immunotherapy and potentially reviving the efficacy of adoptive cell therapies. Future success in this emerging field will hinge on several critical advancements. First, the field must fully embrace the spatial and temporal heterogeneity of metabolic programs within the GC TME, utilizing advanced tools like spatial metabolomics to guide precision targeting. Second, the development of robust predictive biomarkers whether based on metabolite levels, enzymatic expression, or metabolic imaging is essential to identify patient subsets most likely to benefit from specific interventions, moving away from a one-size-fits-all approach. Third, mitigating on-target, off-tumor toxicity through innovative drug delivery systems will be crucial for clinical translation. Finally, optimizing treatment will require sophisticated clinical trial designs that can efficiently test personalized combination regimens.

## Data Availability

Not applicable.

## Abbreviations

Gastric Cancer	GC
Tumor Microenvironment	TME
Cancer-Associated Fibroblasts	CAFs
Tumor-Associated Macrophages	TAMs
Myeloid-Derived Suppressor Cells	MDSCs
Cytotoxic T Lymphocytes	CTLs
Regulatory T Cells	Tregs
Programmed Cell Death Protein 1	PD-1
Programmed Death-Ligand 1	PD-L1
Cytotoxic T-Lymphocyte-Associated Protein 4	CTLA-4
Immune Checkpoint Blockade	ICB
Hypoxia-Inducible Factor 1-Alpha	HIF-1α
Lactate Dehydrogenase A	LDHA
Monocarboxylate Transporters	MCTs
Indoleamine 2,3-Dioxygenase 1	IDO1
Tryptophan 2,3-Dioxygenase	TDO2
Aryl Hydrocarbon Receptor	AhR
Adenosine A2A Receptor	A2AR
Adenosine A2B Receptor	A2BR
Arginase 1	ARG1
Inducible Nitric Oxide Synthase	iNOS
Fatty Acid Synthase	FASN
Fatty Acid Oxidation	FAO
Oxidative Phosphorylation	OXPHOS
Adenosine Triphosphate	ATP
Adenosine Monophosphate	AMP
Reactive Oxygen Species	ROS
Nitric Oxide	NO
Cyclic AMP	cAMP
Peroxisome Proliferator-Activated Receptor Alpha	PPARα
Natural Killer Cells	NK cells
Dendritic Cells	DCs
D-2-Hydroxyglutarate	D-2HG
2-Deoxy-D-Glucose	2-DG
Antibody-Drug Conjugates	ADCs
Chimeric Antigen Receptor T Cells	CAR-T
Single-Cell RNA Sequencing	scRNA-seq
ATP Synthase Membrane Subunit C Locus 2	ATP5MC2
Cluster of Differentiation 39	CD39
Cluster of Differentiation 73	CD73
General Control Nonderepressible 2 Kinase	GCN2
G-Protein Coupled Receptor 81	GPR81
Histone Deacetylases	HDACs
Human Epidermal Growth Factor Receptor 2	HER2
Interferon-Gamma	IFN-γ
Interleukin-6	IL-6
Interleukin-8	IL-8
Interleukin-10	IL-10
Methyltransferase-Like 3	METTL3
Mammalian Target of Rapamycin Complex 1	mTORC1
Nuclear Factor of Activated T Cells 1	NFAT1
Phosphoinositide 3-Kinase Delta	PI3Kδ
Pyruvate Kinase M2	PKM2
Signal Transducer and Activator of Transcription 1	STAT1
Tricarboxylic Acid Cycle	TCA
T Cell Receptor	TCR
Transforming Growth Factor-Beta	TGF-β
Tumor Necrosis Factor-Alpha	TNF-α
Vascular Endothelial Growth Factor	VEGF
YTH N6-Methyladenosine RNA Binding Protein 1	YTHDF1
